# Impact of COVID-19 on Tuberculosis Case Detection and Treatment Outcomes in Sierra Leone

**DOI:** 10.3390/tropicalmed6030154

**Published:** 2021-08-19

**Authors:** Sulaiman Lakoh, Darlinda F. Jiba, Mamadu Baldeh, Olukemi Adekanmbi, Umu Barrie, Alhassan L. Seisay, Gibrilla F. Deen, Robert A. Salata, George A. Yendewa

**Affiliations:** 1 College of Medicine and Allied Health Sciences, University of Sierra Leone, Freetown, Sierra Leone; lakoh2009@gmail.com (S.L.); gibrilladeen1960@yahoo.com (G.F.D.); 2 Connaught Hospital, University of Sierra Leone Teaching Hospitals Complex, Ministry of Health and Sanitation, Freetown, Sierra Leone; darlindajiba.dj@gmail.com (D.F.J.); mbaldeh@gmail.com (M.B.); 3 College of Medicine, University of Ibadan, Ibadan, Nigeria; kemiosinusi@gmail.com; 4 Department of Medicine, University College Hospital, Ibadan, Nigeria; 5 Infectious Disease Research Network, Freetown, Sierra Leone; barrieumu1993@gmail.com; 6 National Tuberculosis and Leprosy Control Program, Ministry of Health and Sanitation, Freetown, Sierra Leone; adonkialans@gmail.com; 7 Department of Medicine, Case Western Reserve University School of Medicine, Cleveland, OH 44106, USA; robert.salata@uhhospitals.org; 8 Division of Infectious Diseases and HIV Medicine, University Hospitals Cleveland Medical Center, Cleveland, OH 44106, USA; 9 Johns Hopkins Bloomberg School of Public Health, Baltimore, MD 21205, USA

**Keywords:** tuberculosis, COVID-19, services utilization, resource-limited settings, Sierra Leone

## Abstract

The COVID-19 pandemic has adversely affected tuberculosis (TB) care delivery in high burden countries. We therefore conducted a retrospective study to assess the impact of COVID-19 on TB case detection and treatment outcomes at the Chest Clinic at Connaught Hospital in Freetown, Sierra Leone. Overall, 2300 presumptive cases were tested during the first three quarters of 2020 (intra-COVID-19) versus 2636 in 2019 (baseline), representing a 12.7% decline. Testing declined by 25% in women, 20% in children and 81% in community-initiated referrals. Notwithstanding, laboratory-confirmed TB cases increased by 37.0% and treatment success rate was higher in 2020 (55.6% vs. 46.7%, *p* = 0.002). Multivariate logistic regression analysis found that age < 55 years (aOR 1.74, 95% CI (1.80, 2.56); *p* = 0.005), new diagnosis (aOR 1.69, 95% CI (1.16, 2.47); *p* = 0.007), pulmonary TB (aOR 3.17, 95% CI (1.67, 6.04); *p* < 0.001), HIV negative status (aOR 1.60, 95%CI (1.24, 2.06); *p* < 0.001) and self-administration of anti-TB drugs through monthly dispensing versus directly observed therapy (DOT) (aOR 1.56, 95% CI (1.21, 2.03); *p* = 0.001) independently predicted treatment success. These findings may have policy implications for DOTS in this setting and suggest that more resources are needed to reverse the negative impact of the COVID-19 pandemic on TB program activities in Sierra Leone.

## 1. Introduction

The outbreak of the coronavirus disease 2019 (COVID-19) pandemic in late December of 2019 has led to an unprecedented global health emergency, resulting in millions of deaths, and placing a severe strain on healthcare systems around the world [1]. In an attempt to address a crisis of an unparalleled scale and proportion, several countries implemented population-wide lockdowns as a key component of broader virus control strategies. Unlike in the global north, the impact of the COVID-19 pandemic in sub-Saharan Africa (SSA) and elsewhere in the global south has been more difficult to assess. Although recent reports have indicated that the pandemic is accelerating in SSA, the region has reported fewer confirmed COVID-19 cases (less than 2% of the global COVID-19 burden) and a significantly lower mortality rate compared with developed countries [1,2,3]. The reasons for this phenomenon—described by some as the “Africa paradox”—remain unclear; however, several explanatory theories have been proposed that put African populations at an advantage, including a younger median age of most African populations [4,5], herd immunity acquired from infections with previous coronaviruses [4,6] and the possibility of shared immunogenicity between *P. falciparum* and SARS-CoV-2 proteins in malaria endemic regions [7].

Despite the seemingly different COVID-19 experience in SSA, a major unintended consequence of widespread lockdowns was the severe disruptions in the continent’s response to its main infectious disease killers, including the tuberculosis (TB) epidemic [8,9]. In 2020, there were 10 million incident TB cases globally, with the Africa region accounting for approximately 25% of TB cases [10]. According to a recent provisional report by the World Health Organization (WHO), an estimated 1.4 million fewer TB cases went undetected and untreated in 2020—a 21% decrease from 2019 that is predicted to result in an additional half a million TB-related deaths in 2020 [11]. In response to this sobering forecast, the WHO has declared the restoration and strengthening of essential TB prevention, case detection and treatment services in endemic settings as a priority global health agenda [11].

Sierra Leone is a high-burden TB country in West Africa, with a TB incidence rate of 295 per 100,000 of the population in 2019 [10]. The National TB and Leprosy Control Program of the Ministry of Health and Sanitation of Sierra Leone offers treatment for all individuals diagnosed with TB across 170 centers in the country, with funding and technical support provided by the Global Fund and other partner agencies [12]. In line with current global trends, the country’s key TB indicators have continued to improve in recent years. Between 2015 and 2019, TB-related deaths decreased by 26%, while treatment coverage rose from 54% to 77%, respectively, putting the country well on track to achieving the WHO End TB Strategy milestones for 2020 [10,12].

The first confirmed cases of COVID-19 in Sierra Leone were reported at the end of March 2020. Similar to other countries in the region, the Government of Sierra Leone declared a state of emergency and implemented a series of population-wide lockdowns starting from early April through June 2020, in an attempt to interrupt the cycle of virus transmission and redirect resources—human and healthcare infrastructure—towards the national COVID-19 pandemic response effort. These actions necessitated the reconfiguration of the prevailing models of TB care delivery to support the new social distancing measures. Specifically, these changes took the form of reductions in the frequency of clinic visits to minimize the risk of COVID-19 exposure and switching from directly observed therapy (DOT)—the traditional model of TB treatment delivery in this setting—to once-monthly dispensing of anti-TB drugs for self-administration at home.

The recent provisional data analysis undertaken by the WHO has provided some insight into the impact of the lockdowns on TB care delivery in Sierra Leone. In 2020, there were an estimated 2000 fewer TB notifications countrywide—a 11.3% decrease from 2019 [11]. However, the full effects of the pandemic on TB care in the country remain uncertain. In this study, we aimed to examine the impact of the COVID-19 pandemic on TB case detection and treatment outcomes at the largest TB treatment center in Freetown, Sierra Leone.

## 2. Materials and Methods

### 2.1. Study Design, Setting and Population

We conducted a retrospective study of patients who sought TB care (case detection and anti-TB treatment) at the Chest Clinic at Connaught Hospital in Freetown, Sierra Leone during 2019 (baseline) and 2020 (intra-COVID-19 period). Connaught Hospital is the national referral health center and is part of the University of Sierra Leone Teaching Hospitals Complex, the main teaching affiliate of the College of Medicine and Allied Health Science of the University of Sierra Leone. The Chest Clinic at Connaught Hospital provides TB diagnostic and treatment services and has the largest DOT program in Sierra Leone. Patients of all ages were in the study.

### 2.2. Data Collection, Definitions and Clinic Procedures

Demographic and clinical data were extracted from the medical records of clinic attendees. Presumptive TB was defined as presenting with cough and constitutional B symptoms (fever, night sweats and weight loss) suggestive of active TB infection and lasting for a minimum of two weeks. Laboratory confirmed TB was defined as having at least one sputum test with a positive acid-fast bacillus (AFB) and/or positive Xpert MTB RIF test. Clinically diagnosed TB was defined as having a negative sputum AFB or Xpert MTB RIF test in the presence of compatible clinical symptoms and suggestive chest radiographic findings, as determined by the clinic physician.

Mode of referral was assigned based on initial presentation to the Chest Clinic for evaluation. Self-reporting was defined as presenting directly to the Chest Clinic with symptoms suggestive of TB. Community-initiated cases were defined as presenting on the advice of family members or peers in the community, while health facility-based referrals were defined as presenting from another clinic or health facility.

Once a diagnosis of active TB was made, appropriate anti-TB treatment was commenced, starting with a four-drug regimen consisting of rifampicin, isoniazid, pyrazinamide and ethambutol for two months, followed by a two-drug regimen consisting of isoniazid and rifampin for at least six months. Patients with rifampicin-resistant (RR) TB were referred to the drug-resistant (DR) TB treatment center, situated 10 km away, for further evaluation and management.

Tuberculosis treatment outcomes were defined in accordance with the WHO reporting framework for TB [13]. Treatment success was defined as the sum total of patients who achieved microbiologic cure (negative AFB or Xpert MTB RIF) or completed at least 6 months of anti-TB treatment, whereas patients who were lost to follow up, transferred, experienced treatment failure or died without any other obvious cause of death while on anti-TB treatment were considered as not having achieved treatment success [13].

### 2.3. Reconfiguration of TB Service Delivery in Sierra Leone during the COVID-19 Pandemic

The implementation of a national lockdown in Sierra Leone necessitated the adaptation of TB service delivery procedures to limit exposure to COVID-19. During the months of April, May and June of 2020, TB clinic hours at Connaught Hospital were limited. Additionally, at the beginning of each of these months, patients receiving anti-TB treatment were given a month’s supply of medications for self-administration at home—a departure from the traditional clinic-based DOT model.

### 2.4. Key Time Points and Periods

We defined the year 2019 as the pre-COVID-19 period (baseline) and 2020 as the intra-COVID-19 period. To compare outcomes of interest, the pre-COVID-19 period was further divided into the first (Q1), second (Q2) and third (Q3) quarters, respectively, of 2019; whereas the intra-COVID-19 period was regarded as the corresponding quarters in 2020. Health-seeking behaviors, such as clinic attendance, have been observed to be seasonal in Sierra Leone and may vary widely during the Christmas season; thus, to minimize selection bias, the fourth quarter of the year (Q4) of both the pre- and intra-COVID-19 periods was not included in the study.

### 2.5. Research Questions

Our research questions were the following:

1. What was the impact of the COVID-19 pandemic on TB case detection, i.e., referrals for TB testing and laboratory-confirmed TB case notifications at the Chest Clinic at Connaught Hospital in Freetown, Sierra Leone?
2. How did TB treatment outcomes compare between patients using self-administration of anti-TB medications at home during April to June of 2020 (intra-COVID-19 period), versus patients using clinic-based DOT during April to June of 2019 (baseline)?

### 2.6. Statistical Analysis

Statistical analyses were performed using the SPSS Version 27.0 (Armonk, NY, USA; IBM Corp). Categorical variables were reported as frequencies (percentages). Percentage change in categorical variables were computed using (2020 value − 2019 value) × 100%/2019 value. A positive percentage change in a categorical variable indicated a net increase from 2019, whereas a negative percentage change indicated a net decrease from 2019. Associations between categorical variables were assessed using Pearson’s chi-square or Fisher’s exact tests. Continuous variables were presented as medians (interquartile ranges, IQR) and associations were assessed using the non-parametric independent samples Mann–Whitney U-test. A logistic regression model was used to identify predictors of treatment success. Known or possible factors impacting on TB treatment outcomes were included in the univariate analysis. These included age, gender, type of TB (pulmonary versus extrapulmonary), patient type (newly diagnosed versus relapse), mode of TB diagnosis (laboratory confirmed versus clinically diagnosed), treatment method (DOT versus self-administered therapy through monthly dispensing) and HIV status. All variables were included in the multivariable model regardless of the *p*-value attained in the univariable analysis. Odds ratios (OR) and adjusted odds ratios (aOR) were presented with 95% confidence intervals (CI). In all analyses, statistical significance was set at *p* < 0.05.

### 2.7. Ethical Considerations

Ethics approval was obtained from the Sierra Leone ethics and scientific review committee (approval date 10 December 2020). As a retrospective study, it did not involve the participation of human subjects, and written informed consent was therefore waived. All patient information was de-identified before entry into a password protected spreadsheet that was only accessible to study personnel.

## 3. Results

### 3.1. Comparison of Presumptive and Laboratory-Confirmed TB Cases during the Pre- and Intra-COVID-19 Periods

Table 1 displays the characteristics of presumptive and laboratory-confirmed TB cases during the first three quarters of 2019 (baseline) and 2020 (intra-COVID-19 period), respectively. A total of 2300 referrals for TB testing were registered during Q1–Q3 in 2020, compared with 2636 in 2019, representing a 12.7% decrease in TB referrals. This corresponded to decreases in males and females (2.3% vs. 24.9%, respectively), as well as among children and adults (19.6% vs. 12.3%, respectively). There was a dramatic decline in community-initiated referrals for TB testing (81.3%), whereas self-reporting and health facility-initiated referrals experienced 7.7% and 46.6% increases, respectively, in 2020, compared with 2019.

Despite the overall decrease in presumptive cases during Q1–Q3 of 2020, there were 733 laboratory-confirmed TB cases, compared with 535 laboratory-confirmed TB cases in 2019, representing a 37.0% increase. Overall, the human immunodeficiency virus (HIV) positivity rate was 31.9% in 2019 and 33.9% in 2020, representing a 2% increase.

**Figure 1:**
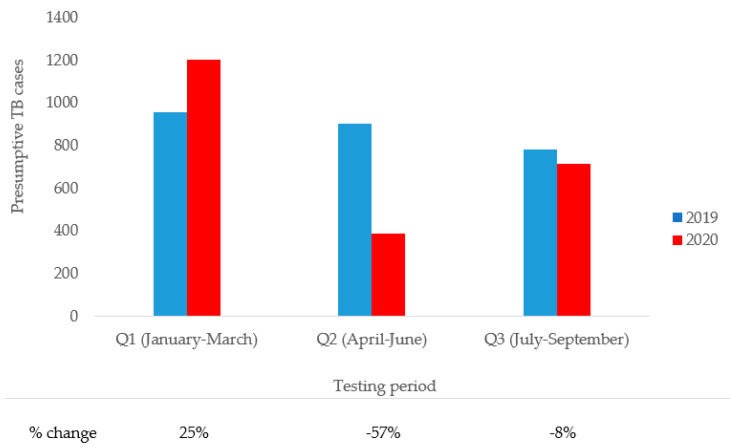
Comparison of presumptive TB cases during the pre- and intra-COVID-19 periods.

Figure 1 compares presumptive TB cases during Q1–Q3 in 2019 and 2020. There was a 25.2% increase in referrals during the first quarter of 2020 compared with 2019. During the second quarter of 2020, there was a 57.1% decrease in referrals, corresponding to the immediate aftermath of the implementation of the lockdown in 2020. The lockdown was eased at the start of July 2020, and by the end of the third quarter of 2020, referrals for TB testing had improved by an 8.2% decrease and were approaching 2019 (baseline) figures.

**Figure 2:**
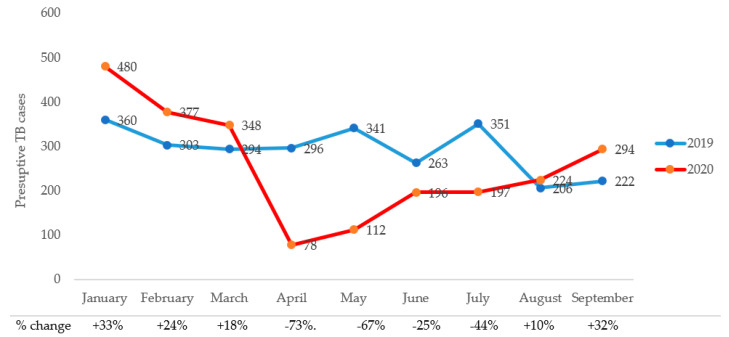
Trends in monthly presumptive TB cases during the pre- and intra-COVID-19 periods.

**Figure 3:**
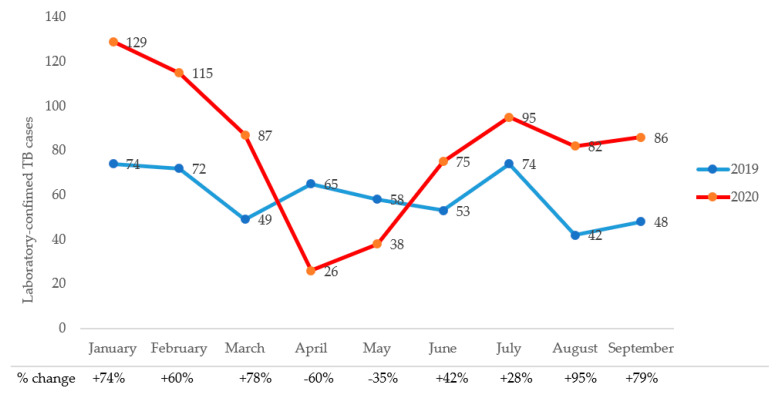
Trends in monthly laboratory-confirmed TB cases during the pre- and intra-COVID-19 periods.

Trends in monthly referrals and laboratory-confirmed TB cases during Q1–Q3 of 2019 and 2020, respectively, are shown in Figure 2. There were more TB referrals during the first quarter of 2020 than 2019. Monthly referrals decreased dramatically from through July of 2020, with the months of April and May 2020 experiencing the most precipitous drops, i.e., 73.5% and 67.2%, respectively, from the corresponding monthly referrals in 2019. Similarly, laboratory-confirmed cases increased by 37% overall in 2020 compared with 2019. However, the lockdowns saw decreases of 60% and 35% in laboratory-confirmed cases in April and May of 2020, respectively compared with the same months in 2019 (Figure 3).

### 3.2. Comparison TB Treatment Outcomes during the Pre- and Intra-COVID-19 Periods

Table 2 compares the characteristics and treatment outcomes of patients who completed TB treatment during April to June of 2019 and 2020, respectively. There was no difference in the distribution of gender (*p* = 0.603), age (*p* = 0.544), patient type (newly diagnosed versus relapsed) (*p* = 0.124), type of TB (pulmonary versus ex-pulmonary) (*p* = 0.873) or HIV status (*p* = 0.339) between the two groups. There were significantly more laboratory-confirmed TB cases in 2020 (*p* = 0.004). Three hundred and fifty-nine (29.7%) patients who completed treatment during the intra-COVID-19 period (April to June 2020) did so by means of self-administration through monthly dispensing.

Overall, there was a higher TB treatment success rate among patients completing treating during April to June of 2020 (55.6% versus 46.7%, *p* = 0.002), with lower rates of loss to follow up (16.3% versus 21.3%, *p* < 0.001) and death (8.9% versus 17.8%, *p* < 0.001), compared with parents completing treatment during April to June of 2019 (pre-COVID-19).

### 3.3. Predictors of TB Treatment Success during the Pre- and Intra-COVID-19 Periods

Multivariate regression analysis (Table 3) found that age < 55 years (aOR 1.74, 95% CI (1.80, 2.56); *p* = 0.005), new TB diagnosis versus relapsed (aOR 1.69, 95% CI (1.16, 2.47); *p* = 0.007), pulmonary TB versus extra-pulmonary (aOR 3.17, 95% CI (1.67, 6.04); *p* < 0.001), HIV negative status(aOR 1.60, 95%CI (1.24, 2.06); *p* < 0.001) and self-administration of anti-TB drugs through monthly dispensing versus clinic-based DOT (aOR 1.56, 95% CI (1.21, 2.03); *p* = 0.001) independently predicted treatment success. 

## 4. Discussion

This study assessed the impact of the COVID-19 pandemic on TB service delivery and treatment outcomes at the largest treatment center in Sierra Leone. We observed an overall 12.7% decrease in presumptive TB cases during the first three quarters of 2020 compared with 2019. Children and women were the most negatively impacted, experiencing an estimated 20% and 25% decline in testing for TB, respectively. The most dramatic decline in presumptive cases occurred in the months of April and May of 2020, coinciding with the immediate aftermath of the implementation of the nationwide lockdowns due to COVID-19. During these months, there was on average a 70% drop in community-initiated referrals. Our observations are consistent with recent studies from Nigeria, Ethiopia, Malawi, Zimbabwe and elsewhere in SSA [14,15,16,17,18,19]. While these findings have been attributed largely to the direct effects of the COVID-19 lockdowns, Thakur et al. and others [15,16,17] have suggested that other factors unrelated to the COVID-19 pandemic may be contributory. In Sierra Leone and other low- and middle-income countries (LMICs) in SSA, low health literacy rates; age- and gender-related differences in risk perception and care-seeking behavior; high out-of-pocket formal and informal costs of services; and transportation constraints are important barriers that commonly result in sub-optimal access and utilization of health services [20,21].

Although there were fewer presumptive cases that underwent testing in 2020, there was a 37% increase in laboratory-confirmed TB cases compared with 2019. Similar findings have been reported in Malawi and Zimbabwe during the COVID-19 pandemic [15,16]. Before 2017, the Chest Clinic at Connaught Hospital relied exclusively on sputum testing for acid-fast bacilli (AFB) by microscopy to confirm TB. As a result of WHO endorsement, however, TB treatment facilities in Sierra Leone and other LMICs have been gaining access to improved diagnostics in recent years. Of note, Xpert MTB RIF testing was piloted in the country in 2016 and scaled-up in 2019 to improve diagnostic capacity and help address growing concerns around the rise of drug-resistant TB in SSA [22]. In our study, a laboratory confirmed case was defined as having at least one sputum test with a positive AFB and/or positive Xpert MTB RIF test, which could partly explain the increase in laboratory-confirmed tests. It is also possible that despite shortened TB clinic hours during the COVID-19-related lockdowns in 2020, laboratory operations may have remained relatively unaffected at this tertiary health facility.

Despite initial concerns that the COVID-19 pandemic would lead to poor TB treatment outcomes, treatment success rates were significantly higher during the intra-COVID-19 period (April to June 2020) compared with the corresponding period in 2019. These figures represent a modest decline from the overall annual TB treatment success rate of 60% that we had previously reported at this facility in 2017 [23] but remain lower than the national treatment success rate of 89% reported in 2019 [10]. Additionally, we recorded significantly lower rates of loss to follow up and TB-related deaths during the COVID-19 pandemic. Our analysis of factors associated with treatment outcomes during the pre- and intra-COVID-19 periods identified the usual predictors of TB treatment success that have been described in the literature—i.e., younger age, newly diagnosed versus relapsed TB, pulmonary TB and HIV negative status [24,25,26]; however, the comparative success of monthly dispensing of anti-TB medications for self-administration at home over the traditional DOT model, though not entirely surprising, was an important and noteworthy finding in this setting.

Directly observed therapy is the most widely used model in delivering TB treatment in endemic countries. Various versions of DOT have been endorsed by the WHO since 1994 as the standard of care for both drug-susceptible and drug-resistant TB, with high rates of cure and treatment completion (>80%), high treatment adherence rates and the ability to directly measure program performance often perceived by policymakers as major strengths [27,28]. However, a 2015 Cochrane review of 11 randomized or quasi-randomized clinical trials comparing DOT to self-administration (N = 5662, with nine LMICs represented) reported low TB cure and treatment completion rates with either treatment approach (41% to 78%); importantly, DOT was not shown to substantially improve these treatment indicators [29].

The high financial cost and logistical and human-resource implications of DOT have led to calls by public health and policymakers to consider alternative approaches to TB care delivery [30,31]. Thus, hybrid approaches using a combination of DOT and self-administration of anti-TB medications through monthly dispensing after the initial induction phase of TB treatment may be useful in Sierra Leone and other regions, especially during public health emergencies requiring social distancing measures, such as that which occurred during the West African Ebola outbreaks of 2014–2016. However, larger studies are needed to confirm our findings.

Our study had a few limitations worthy of mentioning. Firstly, the study design was retrospective and relied on the accuracy of clinic record keeping, which may have suffered during the COVID-19 lockdowns. Secondly, the study was restricted to a single urban referral treatment center in Freetown, which provides care to sicker patients; hence, our findings may not be readily generalizable to the wider population of Sierra Leone. Analysis of programmatic data will provide more information on the full impact of COVID-19 on TB control efforts in the country. Thirdly, we only accessed treatment outcomes during the second quarters of 2019 and 2020; it is remains uncertain whether the improvement in treatment outcomes observed during intra-COVID-19 period with self-administration would have persisted if this model of TB treatment for a more extended period. Despite these shortcomings, our study could have important policy implications for TB control efforts in this high burden setting in Sierra Leone.

## 5. Conclusions

In summary, the COVID-19 pandemic negatively impacted TB care delivery at the largest treatment center in Sierra Leone, with fewer presumptive cases referred to testing in 2020 compared to 2019. Notwithstanding, treatment success rates were higher in 2020. Among others, self-administration of anti-TB drugs independently predicted treatment success, a finding that may have policy implications for TB control efforts in this high burden country. More resources are needed to strengthen essential TB services to help reverse the negative impact of the COVID-19 pandemic on TB program activities in Sierra Leone.

## Figures and Tables

**Table 1:** Comparison of referrals for TB testing and laboratory confirmed TB cases during the pre- and intra-COVID-19 periods.

Characteristics	2019 (Baseline)			2019 Totals	2020 (Intra-COVID-19)			2020 Totals	% Change in 2019 and 2020 Totals	*p*-Value
	Q1 (January–March)	Q2 (April–June)	Q3 (July–September)		Q1 (January–March)	Q2 (April–June)	Q3 (July–September)			
**Presumptive cases**	957	900	779	2636	1199	386	715	2300	−12.7%	
**Gender**										
Male	504	480	432	1416	709	247	428	1384	−2.3%	<0.001
Female	453	420	347	1220	490	139	287	916	−24.9%	
**Age category**										
Children (≤15 years)	62	71	35	168	102	11	22	135	−19.6%	<0.001
Adults (>15 years)	895	829	744	2468	1097	375	693	2165	−12.3%	
**Mode of referral**										
Self-reporting	219	229	160	608	367	104	184	655	+ 7.7%	<0.001
Health facility	322	348	320	990	770	238	443	1451	+46.6%	
Community-initiated	416	323	299	1038	62	44	88	194	−81.3%	
**HIV positivity rate**	31.7%	30.2%	34.3%	31.9%	32.2%	30.8%	38.3%	33.9%	+2.0%	0.011
**Laboratory-confirmed TB**	195	176	164	535	331	139	263	733	+37.0%	<0.001

Q, quarter; TB, tuberculosis.

**Table 2:** Comparison of TB treatment outcomes during the pre- and intra-COVID-19 periods.

Characteristics	Notified TB Cases (N = 1208)	Pre-COVID-19 (April–June 2019) (N = 613)	Intra-COVID-19 (April–June 2020) (N = 595)	*p*-Value
**Gender**				
Male	756 (62.6)	388 (63.3)	368 (61.8)	0.603
Female	452 (37.4)	225 (36.7)	227 (38.2)	
**Age**, *years*				
≤24	300 (24.8)	151 (24.6)	149 (25.0)	0.544
25–34	346 (28.6)	186 (30.3)	160 (26.9)	
35–44	255 (21.1)	131 (21.4)	124 (20.8)	
45–54	174 (14.4)	80 (13.1)	94 (15.8)	
≥55	133 (11.0)	65 (10.6)	68 (11.4)	
**Patient type**				
Newly diagnosed	1069 (88.5)	551 (89.9)	518 (87.1)	0.124
Relapsed	139 (11.5)	62 (10.1)	77 (12.9)	
**Type of tuberculosis**				
Pulmonary	1152 (95.4)	584 (95.3)	568 (95.5)	0.873
Extrapulmonary	56 (4.6)	29 (4.7)	27 (4.5)	
**Mode of diagnosis**				
Laboratory confirmed	720 (59.6)	341 (55.6)	379 (63.7)	0.004
Clinical diagnosis	488 (40.4)	272 (44.4)	216 (36.3)	
**HIV status**				
Positive	425 (35.2)	206 (33.6)	219 (36.8)	0.399
Negative	752 (62.3)	389 (63.5)	363 (61.0)	
Unknown	31 (2.6)	18 (2.9)	13 (2.2)	
**Treatment method**				
Directly Observed Therapy	849 (70.3)	613 (100)	236 (39.7)	<0.001
Monthly dispensing for self-administration	359 (29.7)	-	359 (29.7)	
**WHO treatment outcomes**				
Cured	384 (31.8)	176 (28.7)	208 (35.0)	<0.001
Completed	233 (19.3)	110 (17.9)	123 (20.7)	
Lost to follow up	227 (18.8)	130 (21.3)	97 (16.3)	
Transferred/not evaluated)	193 (16.0)	83 (13.5)	110 (18.5)	
Failed treatment	9 (0.7)	5 (0.8)	4 (0.7)	
Died	162 (13.4)	109 (17.8)	53 (8.9)	
Treatment success *				
Yes	617 (51.1)	286 (46.7)	331 (55.6)	0.002
No	591 (48.9)	327 (53.3)	264 (44.4)	

* Treatment success was defined as the sum of WHO treatment outcomes of cure and completed, whereas the sum of patients who were lost to follow up, transferred, experienced treatment failure and died was classified as not having achieved treatment success.

**Table 3:** Predictors of TB treatment success during the pre- and intra-COVID-19 periods.

Characteristics	Treatment Success		Univariate Analysis		Multivariate Analysis	
	Yes (N = 617)	No (N = 591)	Crude OddsRatio (95% Confidence Interval)	*p*-Value	Adjusted Odds Ratio (95% Convidence Interval)	*p*-Value
**Gender**						
Male	376 (60.9)	380 (64.3)	0.87 (0.69–1.10)	0.228	0.78 (0.61–1.00)	0.053
Female	241 (39.1)	211 (35.7)	Reference		Reference	
**Age**, *years*						
<55	565 (91.6)	510 (86.3)	1.73 (1.19–2.49)	0.003	1.74 (1.08–2.56)	0.005
≥55	52 (8.4)	81 (13.7)	Reference		Reference	
**Patient type**						
Newly diagnosed	532 (86.2)	537 (90.9)	1.59 (1.11–2.28)	0.012	1.69 (1.16–2.47)	0.007
Relapsed	85 (13.8)	54 (9.1)	Reference		Reference	
**Type of tuberculosis**						
Pulmonary	603 (97.7)	549 (92.9)	3.30 (1.78–6.10)	<0.001	3.17 (1.67–6.04)	<0.001
Extrapulmonary	14 (2.3)	42 (7.1)	Reference		Reference	
**Mode of diagnosis**						
Laboratory-confirmed	395 (64.0)	325 (55.0)	1.46 (1.16–1.83)	0.001	1.14 (0.88–1.47)	0.318
Clinical diagnosis	222 (36.0)	266 (45.0)	Reference		Reference	
**HIV status**						
Positive	194 (32.1)	231 (40.4)	Reference	0.003	Reference	<0.001
Negative	411 (67.9)	341 (59.6)	1.43 (1.13–1.82)		1.60 (1.24–2.06)	
**Treatment method**						
Directly Observed Therapy	404 (65.5)	445 (75.3)	Reference	<0.001	Reference	0.001
Monthly Dispensing for Self-administration	213 (34.5)	146 (24.7)	1.61 (1.25–2.06)		1.56 (1.21–2.03)	

## Data Availability

The raw de-identified data supporting the conclusions of this article will be made available by the authors, without undue reservation, to any qualified researcher.
